# Supplementary material for: Coral disease prevalence estimation and sampling design
Source: PeerJ. 2018 Dec 3;6:e6006. doi: 10.7717/peerj.6006 (PMC6282945; doi:10.7717/peerj.6006)

**Supplementary Material**

**Coral disease prevalence estimation and sampling design**

Eric Jordán-Dahlgren1*, Adán Guillermo Jordán-Garza2, Rosa Elisa Rodríguez-Martínez1

Suppl. Fig. 1. Plots of Besag’s L used to assess point distribution departure from spatial random point distribution for the **random** point pattern distribution (A). The black solid line shows L(r)-r (Besag’s normalized Ripley’s K), which is an estimate of the number of points per area in a circle of radius r). The plot then shows the expected point distribution density as a function of distance (r). The dotted red line at zero corresponds to a complete spatial randomness distribution at any spatial range. Deviations from this line suggest clustering if positive and over-dispersion if negative at a given spatial range. Significant deviations occur when the L line falls outside the 95% confidence band (grey band) obtained by Monte Carlo simulations. B) Random transmission diseased point distribution within the random point pattern. C) Nearest neighbor disease transmission point distribution within the random point pattern.


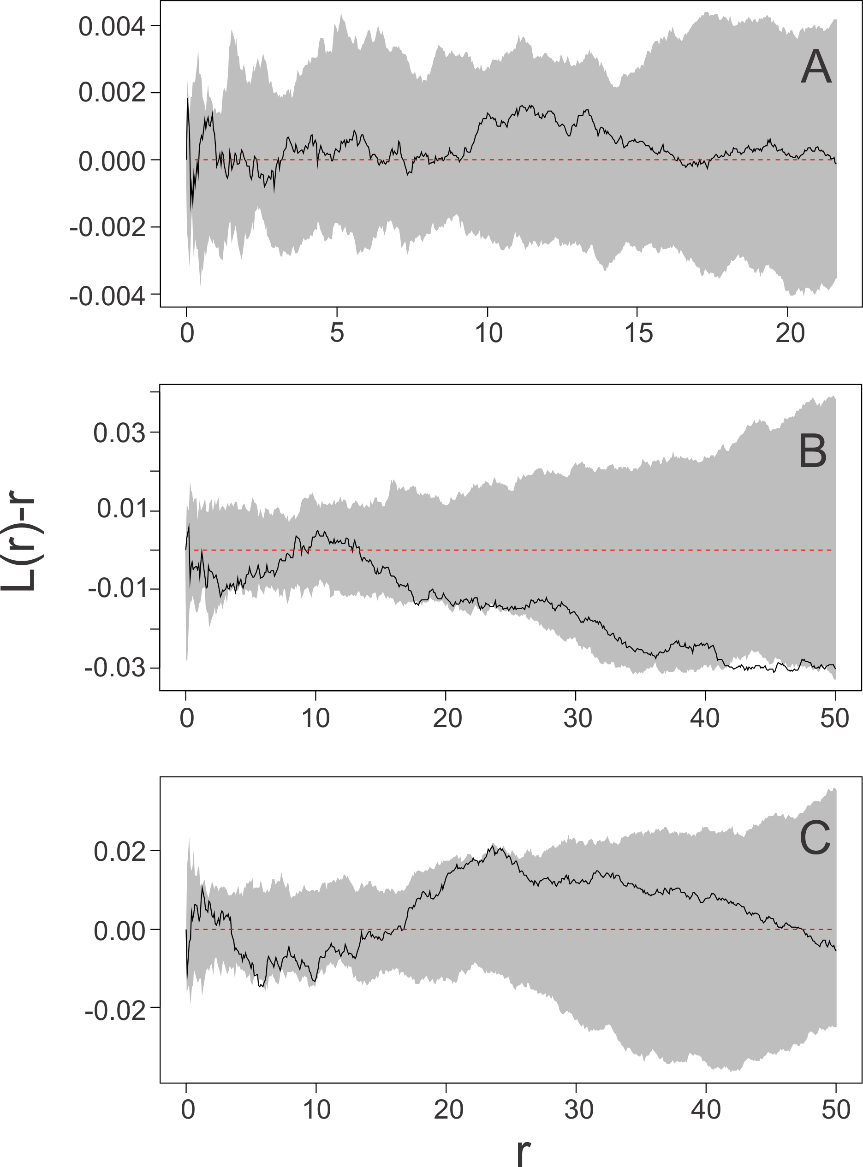


Suppl. Fig. 2. Plots of Besag’s L used to assess point distribution departure from spatial random point distribution for the **clustered** point pattern distribution (A). The black solid line shows L(r)-r (Besag’s normalized Ripley’s K), which is an estimate of the number of points per area in a circle of radius r). The plot then shows the expected point distribution density as a function of distance (r). The dotted red line at zero corresponds to a complete spatial randomness distribution at any spatial range. Deviations from this line suggest clustering if positive and over-dispersion if negative at a given spatial range. Significant deviations occur when the L line falls outside the 95% confidence band (grey band) obtained by Monte Carlo simulations. B) Random transmission diseased point distribution within the random point pattern. C) Nearest neighbor disease transmission point distribution within the random point pattern.


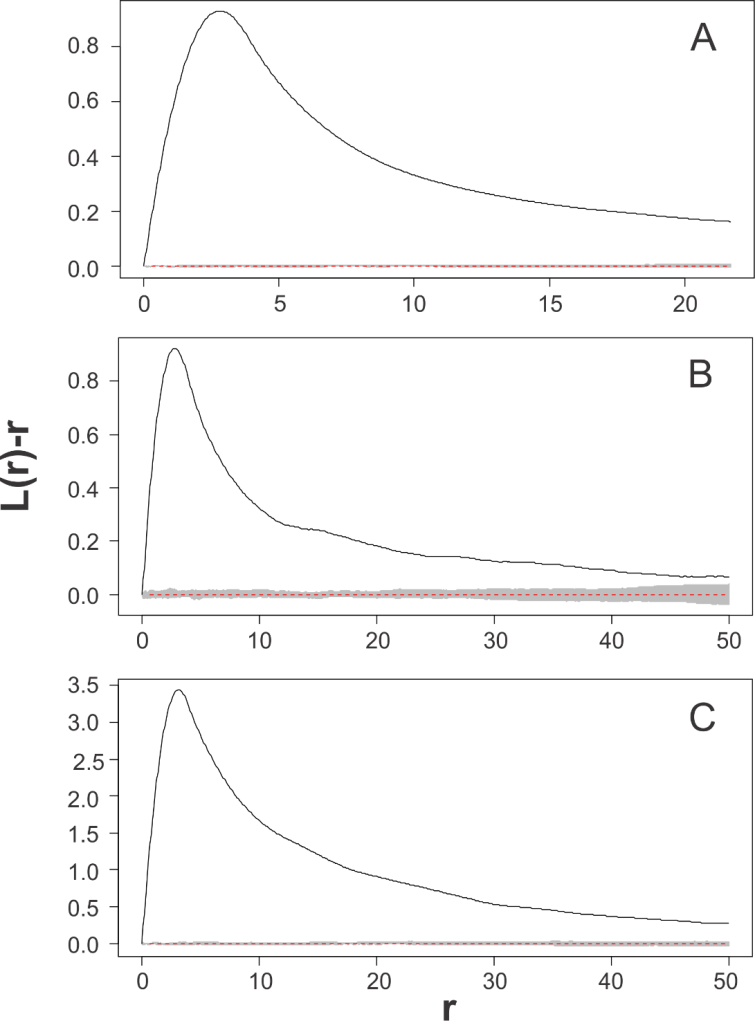


Suppl. Fig. 3. Plots of Besag’s L used to assess point distribution departure from spatial random point distribution for the **over-dispersed** point pattern distribution (A). The black solid line shows L(r)-r (Besag’s normalized Ripley’s K), which is an estimate of the number of points per area in a circle of radius r). The plot then shows the expected point distribution density as a function of distance (r). The dotted red line at zero corresponds to a complete spatial randomness distribution at any spatial range. Deviations from this line suggest clustering if positive and over-dispersion if negative at a given spatial range. Significant deviations occur when the L line falls outside the 95% confidence band (grey band) obtained by Monte Carlo simulations. B) Random transmission diseased point distribution within the random point pattern. C) Nearest neighbor disease transmission point distribution within the random point pattern.


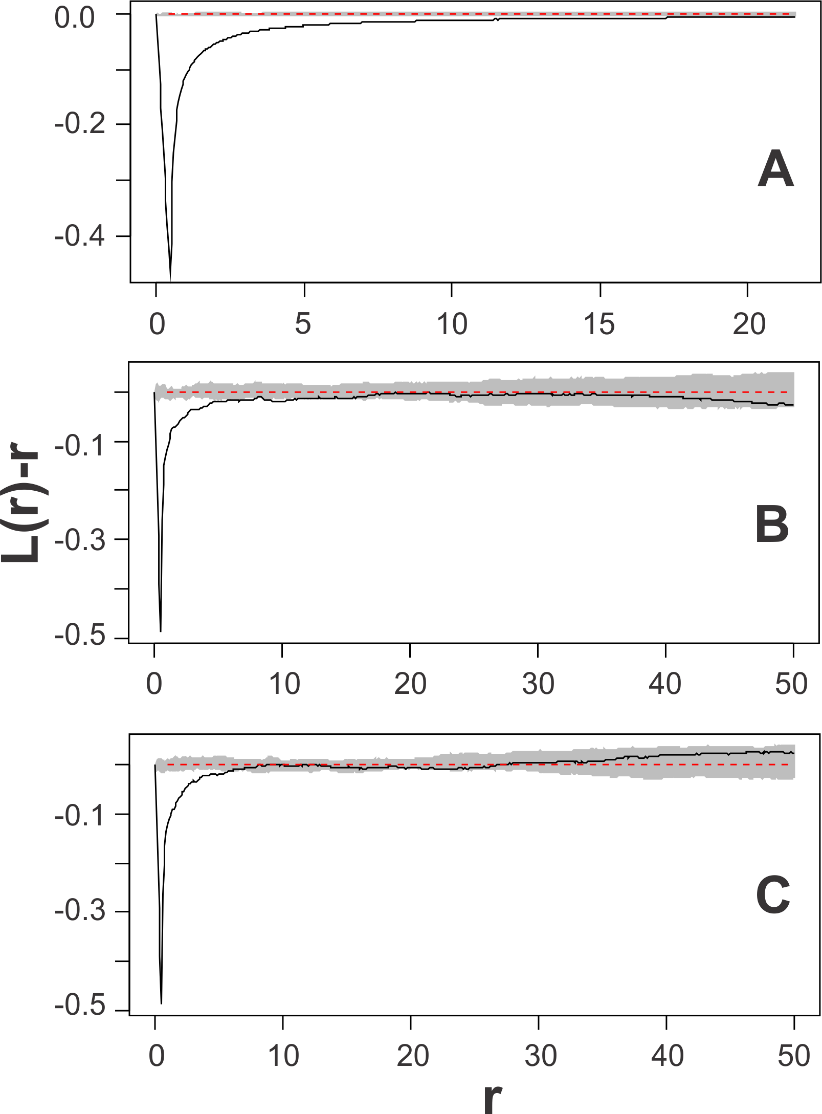

Supplement: Supplemental Information 1 — Plots of Besag’s L used to assess point distribution departure from spatial random point distribution. [file peerj-06-6006-s001.docx]
