# Supplementary material for: Coral disease prevalence estimation and sampling design
Source: PeerJ. 2018 Dec 3;6:e6006. doi: 10.7717/peerj.6006 (PMC6282945; doi:10.7717/peerj.6006)
Supplement: Supplemental Information 2 — Coral reef ecology sampling protocols that are commonly used to estimate coral disease prevalence reported in the literature. [file peerj-06-6006-s002.docx]

| Protocol used by | Study Area | Probabilistic design | Sampling | | | | Details | Reference |
| --- | --- | --- | --- | --- | --- | --- | --- | --- |
|  |  |  | **Stations per site or reef zone** | **Units #** | **Unit Size (m)** | **Site Area (m^2^)** |  |  |
| AGRRA,  Healthy Reefs | Various | No | 1 | 6 | 10x1 | 60 | Habitat-type stratification. Advises six transects or 50 corals. | http://www.agrra.org/training-tools/agrra-method/ |
| CRTR | Various | No | 1 | 3 | 10x2 | 60 | Advises at least three haphazardly placed transects in areas of high coral cover. | Raymundo et al. 2008 |
| CREMP | Florida | No | 4 | 22 | 22x2 | 968 | Habitat stratification; one site has four stations. In large reef areas, stations are set in parallel formations, several meters apart. In limited habitat area the station was placed on available habitat of interest. | http://myfwc.com/research/habitat/coral/cremp/site-selection-monitoring/ |
| AIMS | Australia | No | 1 | 5 | 50x2 | 500 | Habitat-type stratification. Uses five transects per site. | http://www.aims.gov.au/334 |
| Reef Check | Various | No | 1 | 4 | 20x5 | 400 | Four transects per site laid one after the other, separated by 5 m intervals. | http://www.reefcheckaustralia.org/files/documents/442/rca_methods_2013.pdf |
| Raymundo et al. | Philippines | No | 1 | 3 | 20x2 | 120 | Three transects per site | Raymundo et al. 2005 |
| Richardson and Voss | Florida | No | 1 | 1 | 10m radius circle | 314 | One circle per site, one to nine sites per reef. | Richardson & Voss 2005 |
| Kaczmarsky | Philippines | No | 1 | 6 | 10x2 | 120 | Six transects in haphazardly selected sites. Transects were placed parallel to each other or in a straight line. | Kaczmarsky 2006 |
| Haapkylä et al. | Wakatobi National Park | No | 1 | 3 | 20x4 | 240 | Habitat-type stratification. Three transects laid by zone. | Haapkylä et al. 2007 |
| Lamb and Willis | Australia | No | 1 | 6 | 15x2 | 180 | Six transects per site | Lamb & Willis 2011 |
| Aeby et al. | Hawaii | No | 1 | 2 | 25x6 | 300 | Two transects per site. | Aeby et al. 2011 |
| Ruiz-Moreno et al. | Various | No | 2 - 4 | 3-39 | 2.5x1 10x2 20x1 20x2 25x2 | 60 - 1200 | Varied: Three 20x1m transects; 39 2.5x1 m transects; two stations, six 20x2m transects each; two stations, five transects 10x2 m each; four stations, six 25x2 m transects each. | Ruiz-Moreno et al. 2012 |
| Smith et al. | Florida | Yes limited | 1 | 5 | 25x0.4 | 50 | Habitat-type stratification. Two to 13 transects (average 5) depending on the size of the survey area (0.68 to 42.5 km^2^, average: 11 km^2^). | Smith et al. 2011 |
